# Tumor-Penetrating Delivery of siRNA against TNFα to Human Vestibular Schwannomas

**DOI:** 10.1038/s41598-017-13032-9

**Published:** 2017-10-10

**Authors:** Yin Ren, Jessica E. Sagers, Lukas D. Landegger, Sangeeta N. Bhatia, Konstantina M. Stankovic

**Affiliations:** 1 Eaton Peabody Laboratories, Massachusetts Eye and Ear, 243 Charles Street, Boston, MA 02114 USA; 2 Department of Otolaryngology, Massachusetts Eye and Ear, 243 Charles Street, Boston, MA 02114 USA; 3 Department of Otolaryngology, Harvard Medical School, 25 Shattuck St, Boston, MA 02115 USA; 4 Koch Institute for Integrative Cancer Research, Massachusetts Institute of Technology, 77 Massachusetts Avenue, Cambridge, MA 02139 USA; 5 Harvard Program in Speech and Hearing Bioscience and Technology, 25 Shattuck Street, Boston, MA 02115 USA; 6 Department of Otolaryngology, Medical University of Vienna, Waehringer Guertel 18-20, 1090 Vienna, Austria; 7 Institute for Medical Engineering and Science, 77 Massachusetts Avenue, Cambridge, MA 02139 USA; 8 Department of Electrical Engineering and Computer Science, MIT, 77 Massachusetts Avenue, Cambridge, MA 02139 USA; 9 Department of Medicine, Brigham and Women’s Hospital, 75 Francis Street, Boston, MA 02115 USA; 10 Howard Hughes Medical Institute, 4000 Jones Bridge Road, Chevy Chase, MD 20815 USA

## Abstract

Vestibular schwannoma (VS) is the most common tumor of the cerebellopontine angle, and it typically presents with sensorineural hearing loss. The genomic landscape of schwannoma is complex and many of the molecules implicated in VS pathogenesis represent targets not amenable to antibody-based or small molecule therapeutics. Tumor-targeted delivery of small interfering RNA (siRNA) therapeutics provides a direct and effective means to interrogate targets while minimizing off-target effects. To establish a preclinical model for therapeutic inhibition of putative targets in VS, archived tumor specimens, fresh tumor cells derived from patients with sporadic VS, and an established schwannoma cell line were screened. Nanoparticles directed by the tumor-homing peptide iRGD were selectively taken up by primary VS cultures *in vitro* via interactions with αvβ3/β5 integrins and neuropilin-1 (NRP-1). Cellular uptake was inhibited by a neutralizing antibody against αv integrin in a dose-dependent manner. When applied to primary VS cultures, iRGD-targeted nanoparticles delivered siRNA directed against TNFα in a receptor-specific fashion to potently silence gene expression and protein secretion. Taken together, our results provide a proof of principle for tumor-targeted, nanoparticle-mediated delivery of siRNA to VS and establish a novel platform for the development and pre-clinical screening of molecular therapeutics against VS.

## Introduction

Vestibular schwannomas (VSs) are the most common tumors of the cerebellopontine angle and the fourth most common intracranial tumors^1^. They arise from Schwann cells lining the vestibular branch of cranial nerve VIII and are associated with significant morbidity including asymmetric sensorineural hearing loss (SNHL) which affects 95% of patients, dizziness, other cranial neuropathies such as facial palsy, and even brainstem compression and hydrocephalus^2^.

To date, the mainstays of therapy for growing VSs include surgical resection and stereotactic radiation, as there are currently no FDA-approved systemic medical therapies to treat VS or ameliorate VS-associated SNHL. Several clinical trials for neurofibromatosis type 2 (NF2)-associated VSs are ongoing^3,4^. Targeted molecular pharmacotherapy to inhibit the VEGF-A signaling pathway in NF2-associated VS has shown early encouraging results; however, substantial side effects such as renal failure prevent widespread clinical use^5,6^.

Work from our laboratory and others has recently begun to uncover new molecular targets implicated in VS tumorigenesis and associated SNHL. For instance, we identified NF-κB as a central regulator in VS proliferation and survival, which can be targeted by clinically relevant inhibitors^7^. We have shown that biological differences exist in gene expression and proteomic profile between sporadic VSs associated with SNHL and VSs associated with good hearing^8,9^. Furthermore, there appears to be a correlation between the degree of SNHL in patients with sporadic VSs and the extent of cellular damage when tumor secretions from these patients are applied to murine cochlear explants^10^. Using a candidate molecule approach, we identified tumor necrosis factor alpha (TNFα) as an ototoxic molecule^11^. However, attempts to target the NF-κB pathway or inhibit TNFα secretion using small molecules yielded only modest benefits *in vitro* ^11^.

As more molecular pathways in VS pathogenesis are discovered, one potential therapeutic strategy is to directly downregulate mediators of tumor growth or ototoxic effects using small interfering RNAs (siRNAs). RNA interference (RNAi) is particularly attractive in addressing gene targets that do not yet have small molecule or antibody-based antagonists. Furthermore, RNAi can be easily scaled up for both *in vitro* screens and pre-clinical studies in animal models. Nevertheless, the effective delivery of intact siRNA molecules into the cytoplasm of tumor cells remains the biggest barrier for clinical translation of RNAi-based therapies^12^. Systemically administered siRNAs must remain stable in circulation and home to the organ of interest while avoiding accumulation in normal tissue. Furthermore, siRNAs must also be selectively taken up by tumor cells, escape endosomal entrapment, and be ultimately incorporated into the RNA-induced silencing complex (RISC) in order to downregulate gene expression.

Cancer nanotechnology offers an attractive solution to the delivery problem. Nanoparticles can be engineered to protect siRNA from degradation by serum nucleases, and to shield the cargo from clearance mechanisms such as the renal glomerular filtration system and the hepatic reticuloendothelial system. Utilizing the enhanced permeability and retention (EPR) effect, nanoparticles preferentially accumulate in tumor tissue as a result of hypervascularity and impaired lymphatic drainage in tumors^13^. By decorating the surface of nanoparticles with affinity ligands such as tumor-targeting peptides, siRNA therapeutics can be preferentially directed to tumor cells of interest via specific cell surface receptor-ligand interactions. This form of synaphic targeting can further improve the micro-distribution of siRNA therapeutics within the tumor by enhancing cellular uptake, retention and subsequent internalization^14^. This strategy has been successfully employed to deliver siRNA therapeutics across the blood-brain barrier (BBB)^15,16^. More recently, a new class of tumor-penetrating peptides has been discovered, which dramatically enhances the targeting of macromolecules and nanoparticles to tumors^17^. The prototypical peptide, iRGD (internalizing RGD, CRGDR/KGPDC), binds to αvβ3/β5 integrins expressed in tumor cells and tumor-associated vasculature. Subsequent proteolytic cleavage of iRGD unveils the cryptic C-terminal arginine, also known as the CendR domain, which activates a trans-tissue transport pathway by binding to neuropilin-1 (NRP-1)^17–19^. This allows infiltration of macromolecular payloads such as albumin-bound paclitaxel and nanoparticles as the tumor becomes more “leaky”^20,21^. Compared to non-tumor penetrating conventional RGD peptides, iRGD dramatically enhances the accumulation and efficacy of delivered drugs.

Previously, our laboratory has engineered a peptide-based siRNA delivery platform for rapidly credentialing gene targets in a mouse model of disseminated ovarian cancer^22^. The delivery system consists of a tandem peptide containing two domains: a nine-amino acid cyclic peptide that is both tumor-targeting and tissue-penetrating, and an amphipathic domain which interacts with the cellular membrane to mediate escape from endosomal entrapment^23^. When complexed with siRNA directed against an oncogene essential for tumor growth, nanoparticles penetrated tumor parenchyma and delivered siRNA into the cytoplasm of tumor cells to reduce tumor growth and prolong animal survival. We hypothesized that such a peptide-based delivery system could be adapted for siRNA delivery to primary human VS cultures and could modulate the secretion of molecules that may mediate ototoxicity.

In this study, we report the development of a tandem peptide-based siRNA delivery system to target primary human VS cultures *in vitro*. Peptides bearing a tumor-targeting domain (iRGD) and a membrane-translocation domain stably encapsulated siRNAs into nanoparticles. In an NF2-derived cell line and primary human VS cells, there was an overexpression of αvβ3/β5 integrins and NRP-1, cognate receptors for the iRGD targeting peptide. Nanoparticles were selectively taken up into the cytoplasm of tumor cells in a receptor-specific fashion. Furthermore, nanoparticle-mediated delivery of siRNA directed against TNFα resulted in potent reduction of protein levels in VS secretions from tumors associated with SNHL.

## Results

### Design and characterization of tumor-penetrating nanoparticles

**Figure 1:**
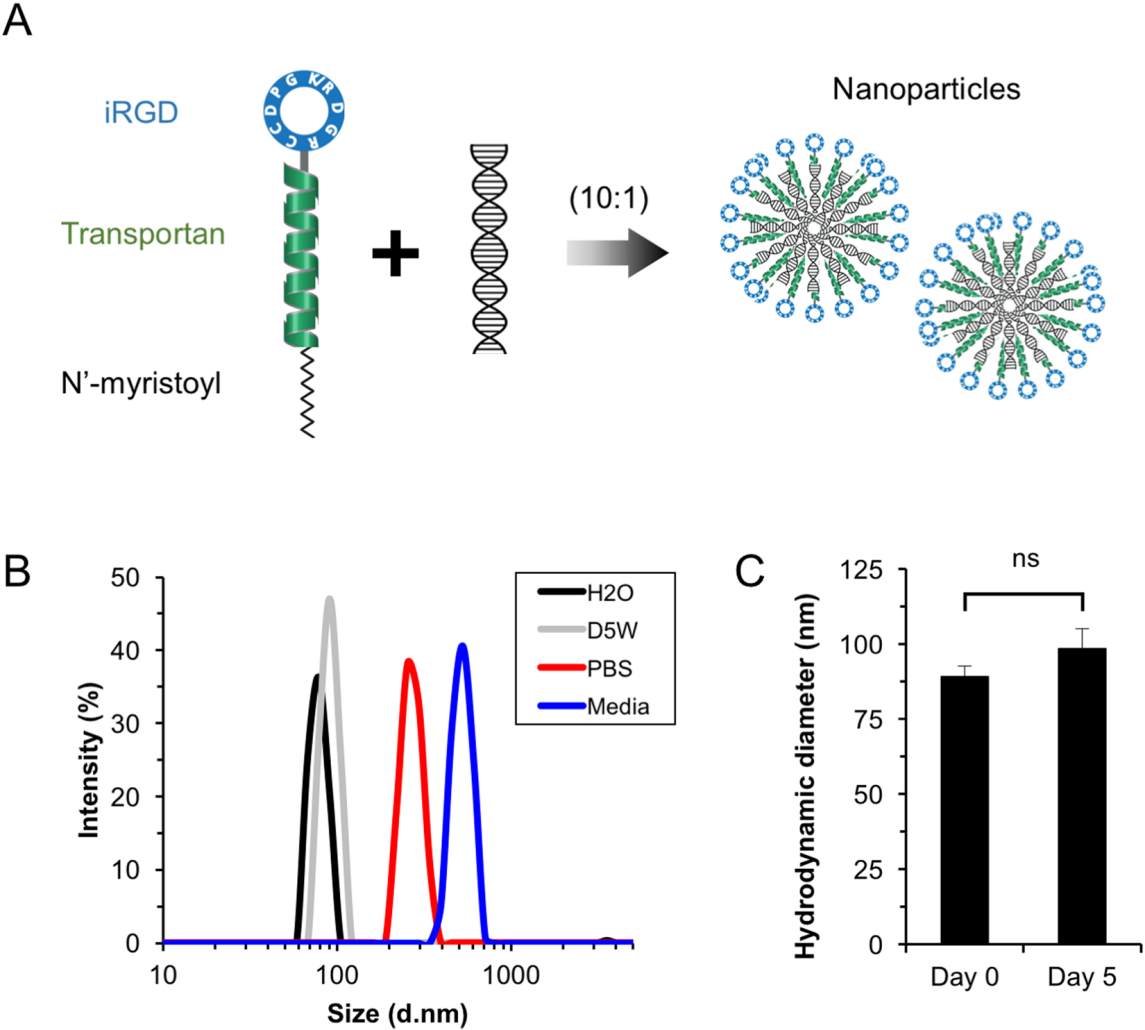
Characterization of iRGD-targeted nanoparticles. (**A**) Schematic of iRGD nanoparticle formation. Tandem peptides consisting of three domains: N-terminal myristoylated acid, cell penetrating peptide domain (Transportan, colored in green), and a cyclic CendR targeting peptide domain (iRGD, CRGDK/RGPDC, colored in blue), is non-covalently mixed with siRNA cargo at a molar ratio of 10:1 (peptide to siRNA) to form nanoparticles as shown. (**B**) Dynamic light scattering (DLS) measurements of hydrodynamic diameter of nanoparticles in pure water (H_2_O, black), 5% dextrose in water (D5W, gray), phosphate buffered saline (PBS, red), or DMEM cell culture media (Media, blue). Representative histograms from 5 independent preparations are shown. (**C**) The hydrodynamic diameter of nanoparticles does not change significantly after incubating for 5 days at room temperature. NS, not significant. Error bars represent standard deviation (n = 5 independent preparations).

Our goal was to engineer a nanoparticle platform that could penetrate the tumor parenchyma, bind selectively to vestibular schwannoma cells, and deliver siRNA into the cytoplasm. We began by investigating the ability of nanoparticles consisting of tandem-peptides complexed with siRNA molecules, previously developed in the laboratory to target human epithelial ovarian cancer cells, to target vestibular schwannoma cells. The peptide bearing the N-terminal myristoylated acid followed by the cell internalization domain Transportan (TP) and the C-terminal targeting domain iRGD (C*RGDK*GPDC) was chosen for this study (Fig. 1A). Transportan is an amphipathic cell penetrating peptide that ubiquitously translocates across the cellular membrane^24^ and condenses siRNAs into nanoparticles^25^. The iRGD peptide consists of the canonical RGD motif which functions as the affinity ligand for αv integrins, and a cryptic C-terminal arginine motif (RGDK), which is unveiled upon proteolytic processing and interacts with NRP-1 to trigger tissue penetration^17,26^. Both αv integrins and NRP-1 have been reported to be overexpressed in many tumor types *in vitro* and *in vivo*, including CNS tumors such as glioblastomas, ependymomas, and vestibular schwannomas^27^. The tandem peptides were admixed with siRNA molecules at a molar ratio of 10:1 (peptide to siRNA), as previous studies have suggested that siRNA molecules can be maximally encapsulated by peptides at this ratio^23^. This resulted in stable sub-100nm nanoparticles based on non-covalent charge-based interactions between the positively-charged amino acid sequence and the negatively-charged nucleic acid backbone. When peptides were mixed with siRNA in pure water or water with 5% dextrose (D5W), the average hydrodynamic diameter was determined to be approximately 80 nm (Fig. 1B). When admixing occurred in phosphate-buffered saline (PBS) or cell culture medium, the resulting particles showed an increase in apparent size to approximately 250–300 nm, likely secondary to some degree of aggregation when electrostatic interactions were neutralized^28^. Control nanoparticles were generated with tandem peptides bearing the non-integrin binding peptide domain (scr, CRGEKGPDC), which shared the same number of amino acids and surface charge as iRGD. Both iRGD and scr control nanoparticles had similar hydrodynamic size distributions, polydispersity indices between 0.25 and 0.3, and zeta potentials between +25 to +35 mV (Supplementary Figure 1). To investigate the long-term stability of peptide-siRNA complexes, freshly prepared nanoparticles were incubated at room temperature over time. The hydrodynamic diameter remained stable after an incubation period of 5 days (Fig. 1C).

### Patient Demographics

Primary vestibular schwannoma (VS) cells were derived from tumor specimens resected from patients with unilateral, sporadic VS per established protocols^29,30^. Control healthy nerve specimens from surgically sacrificed human great auricular nerves (GANs) were also harvested per established protocols. Demographics of the patients used in this study are summarized in Table 1. A combination of patients with good hearing in the ipsilateral ear and patients with poor hearing in the ipsilateral ear were selected for the study. Compared to patients with good hearing, word recognition scores (WR) were significantly lower and ipsilateral pure tone averages (PTA) were significantly greater in patients with poor hearing. In all subjects, the contralateral ear did not exhibit significant SNHL.

**Table 1:** Demographics of patients selected in the study.

Tumor ID	Subject Age	Gender	Tumor Size (mm)	Ipsilateral Ear		Contralateral Ear	
				PTA (dB)	WR (%)	PTA (dB)	WR (%)
VS-1	36	F	9	40	72	18	98
VS-2	30	F	30	28	92	30	100
VS-3	34	F	8	15	94	5	98
VS-4	63	M	15	72	20	10	100
VS-5	46	F	35	50	10	20	96
VS-6	76	M	20	100	0	10	90
VS-7	36	F	23	8	100	5	100
VS-8	28	F	33	40	18	0	98
VS-9	74	F	27	22	74	5	90
VS-10	52	F	27	15	96	10	94
VS-11	51	M	31	25	100	0	76
VS-12	18	F	35	60	100	15	100
VS-13	40	F	32	100	0	100	0
VS-14	23	F	37	58	63	100	0

Tumor ID, subject age, gender, tumor size (mm, largest transverse dimension), pure tone average (PTA, dB), and word recognition score (WR, %) for the ipsilateral and contralateral ears to VS are shown.

### Cell surface expression of receptors for nanoparticle targeting

**Figure 2:**
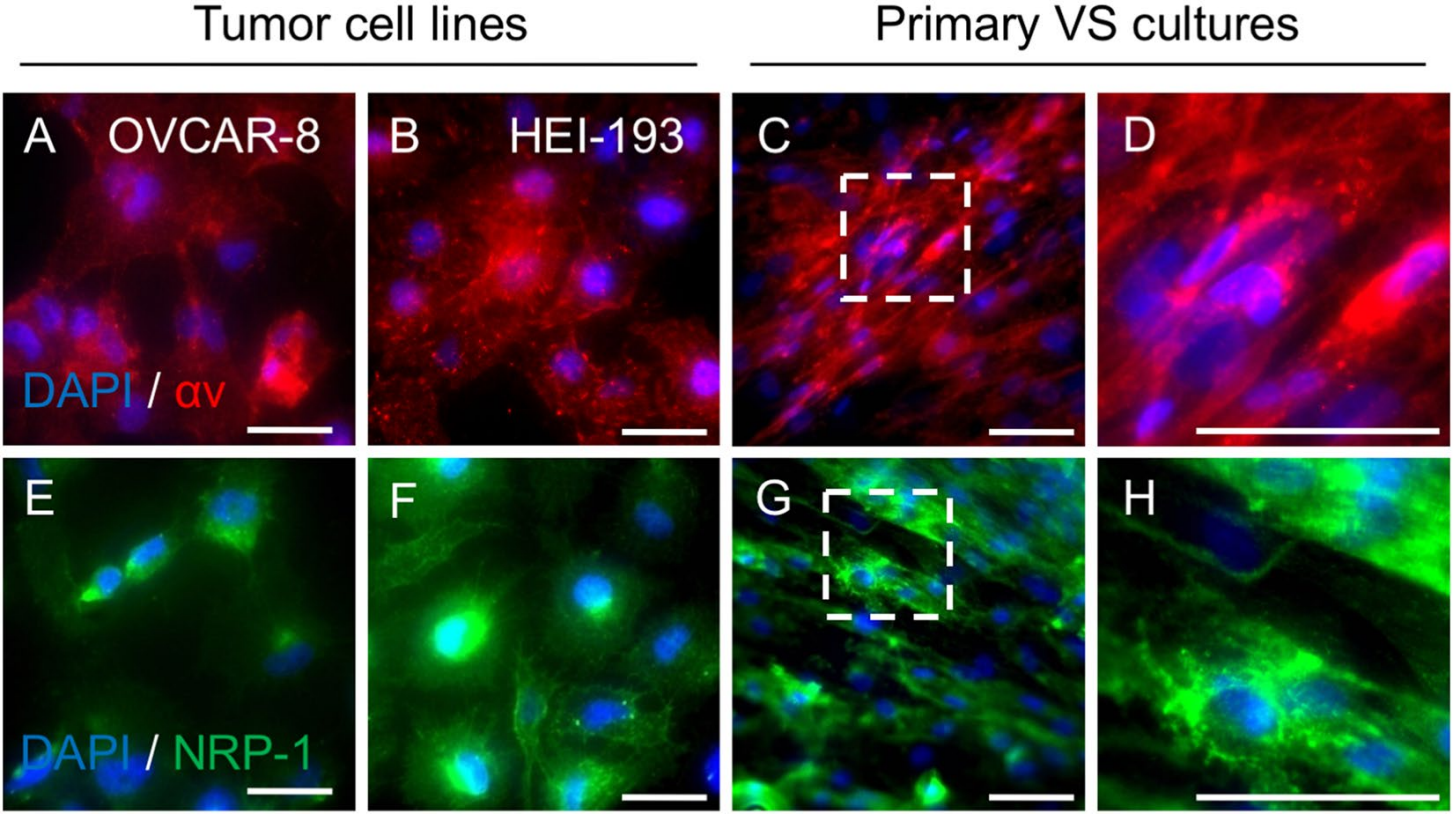
Expression of surface receptors in tumor cell lines and primary human vestibular schwannoma (VS) cultures for iRGD nanoparticle targeting. (**A**–**D**) Immunofluorescence staining of αv integrin (pseudocolored red) in OVCAR-8 cells (**A**), an ovarian cancer cell line known to express αv integrins on the cell surface; HEI-193 cells (**B**), a neurofibromatosis type 2 vestibular schwannoma cell line; and primary human VS culture (**C** and **D**) where (**D**) is an enlarged section from dashed rectangle of (**C**). Nuclei are counterstained with DAPI (pseudocolored blue). For established cell lines, representative images based on 6 independent experiments are shown. For VS cultures, representative images from 3 biological replicates from different tumors are shown. (**E**–**H**) Immunofluorescence staining of Neuropilin-1 (NRP-1, pseudocolored green) in OVCAR-8 cells (**E**), HEI-193 cells (**F**), and primary human VS culture (**G** and **H**) where (**H**) is an enlarged section from dashed rectangle of (**G**). Nuclei are counterstained with DAPI (pseudocolored blue). Six (6) independent experiments were performed for established cell lines, whereas 3 different tumors were analyzed. All scale bars are 100 $\mu$m.

To determine the feasibility of nanoparticle targeting in VS, the receptor expression profile on the tumor cell surface was assessed. Specifically, αv integrin is a part of the family of αvβ3 and αvβ5 integrins which mediates binding of RGD peptide-directed nanoparticles^17,31^. NRP-1 is a receptor for the iRGD peptide, which contains the C-terminal arginine motif and functions as a key mediator for tissue extravasation and tumor penetration^26^. We hypothesized that both VS-derived primary cell cultures and archived tumor specimens from patients with unilateral, sporadic VS overexpressed sufficient amounts of αv integrin and NRP-1. An established human epithelial ovarian cancer cell line (OVCAR-8) known to express both receptors was used as a positive control^22^. In an NF2-derived human schwannoma cell line (HEI-193) and in human primary VS cells from 3 different tumors, αv integrin was overexpressed on the cell surface as determined by immunofluorescence staining (Fig. 2A–D). Consistent with prior reports, integrin expression was mainly observed along the cellular periphery, with clusters of bright aggregations likely contributing to the adherence of cells onto the culturing substrate^32^. Similarly, HEI-193 and human primary VS cells from 3 different tumors expressed NRP-1 on the cell surface at a level similar to that of OVCAR-8 cells (Fig. 2E–H).

**Figure 3:**
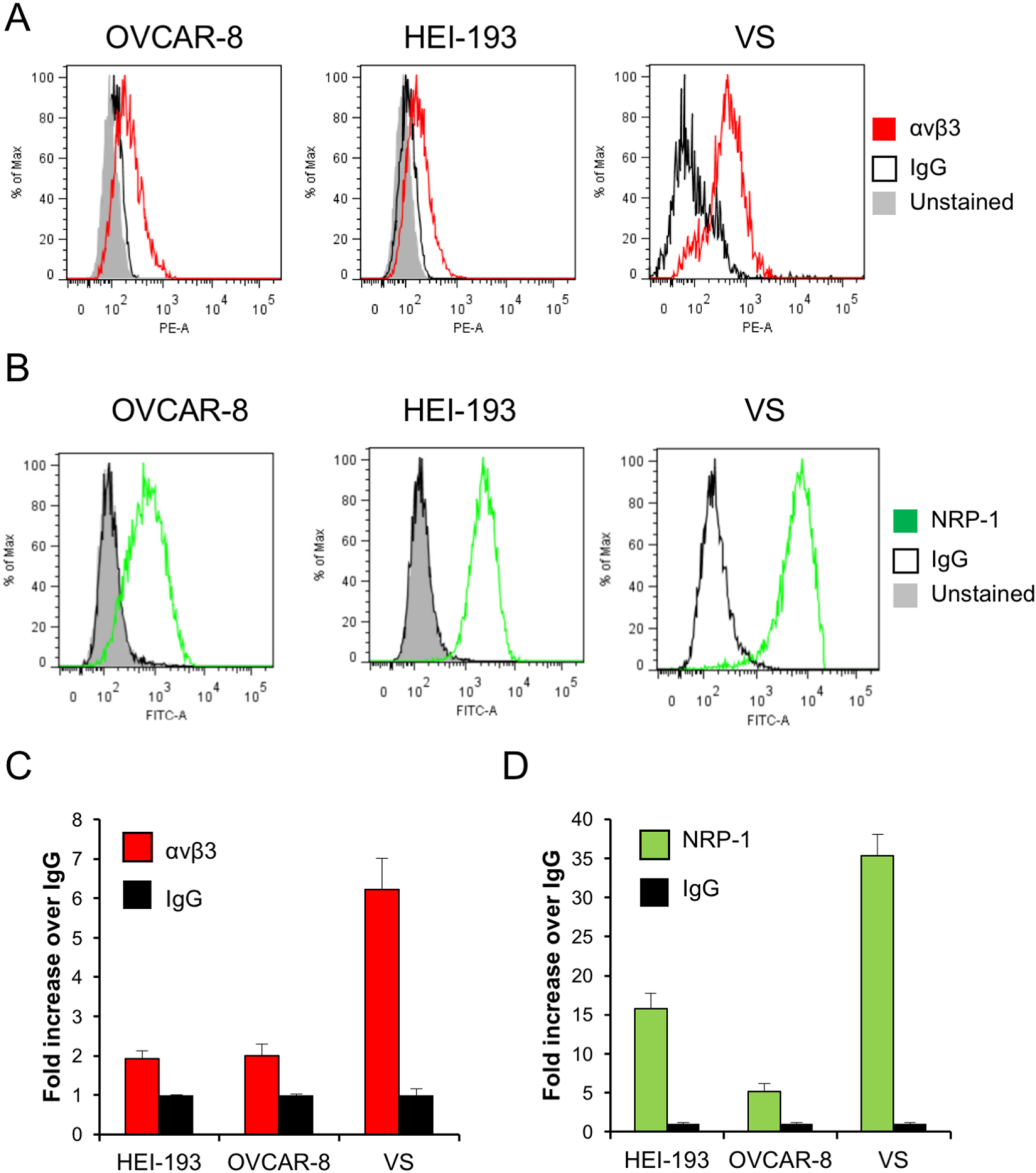
Expression of αv integrins and NRP-1 in cell lines and primary VS cultures as determined by flow cytometry. (**A**) Flow cytometry analysis of αvβ3 integrin expression in OVCAR-8 (left panel), HEI-193 (center panel), and human VS cultures (right panel). Red, αvβ3 integrin antibody. Black, IgG isotype control. Shaded histogram represents unstained OVCAR-8 or HEI-193 cells. Representative histograms from six independent replicates are shown. (**B**) Flow cytometry analysis of NRP-1 surface expression. Red, NRP-1 antibody. Black, IgG isotype control. Shaded represents unstained OVCAR-8 or HEI-193 cells. Representative histograms from six independent replicates are shown. (**C**) Quantification of αvβ3 integrin expression in cell lines and human VS cultures. Red bars, anti-αvβ3 antibody; black bars, IgG control antibody. Error bars represent standard deviation. (n = 6 independent replicates for cell lines, 4 different tumors for VS cultures). (**D**) Quantification of NRP-1 expression. Green bars, anti-NRP-1 antibody; black bars, IgG control antibody. Error bars represent standard deviation. (n = 6 independent replicates for cell lines, 4 different tumors for VS cultures).

To further confirm the presence of αv integrins and NRP-1 at the cell surface and to quantitatively assess the level of expression on a single-cell basis, flow cytometry analyses were performed using polyclonal antibodies directed against αvβ3 and NRP-1. IgG antibodies from the same species were used as isotype controls. Significant levels of cell-surface αvβ3 and NRP-1 were detected in OVCAR-8 ovarian cancer cells, HEI-193 human vestibular schwannoma cells, and human primary VS cells from 4 different tumors (Fig. 3A,B). Interestingly, in comparison to immortalized cancer cell lines, primary VS cells freshly harvested from patients exhibited approximately two- to three-fold higher levels of receptor expression (Fig. 3C,D).

**Figure 4:**
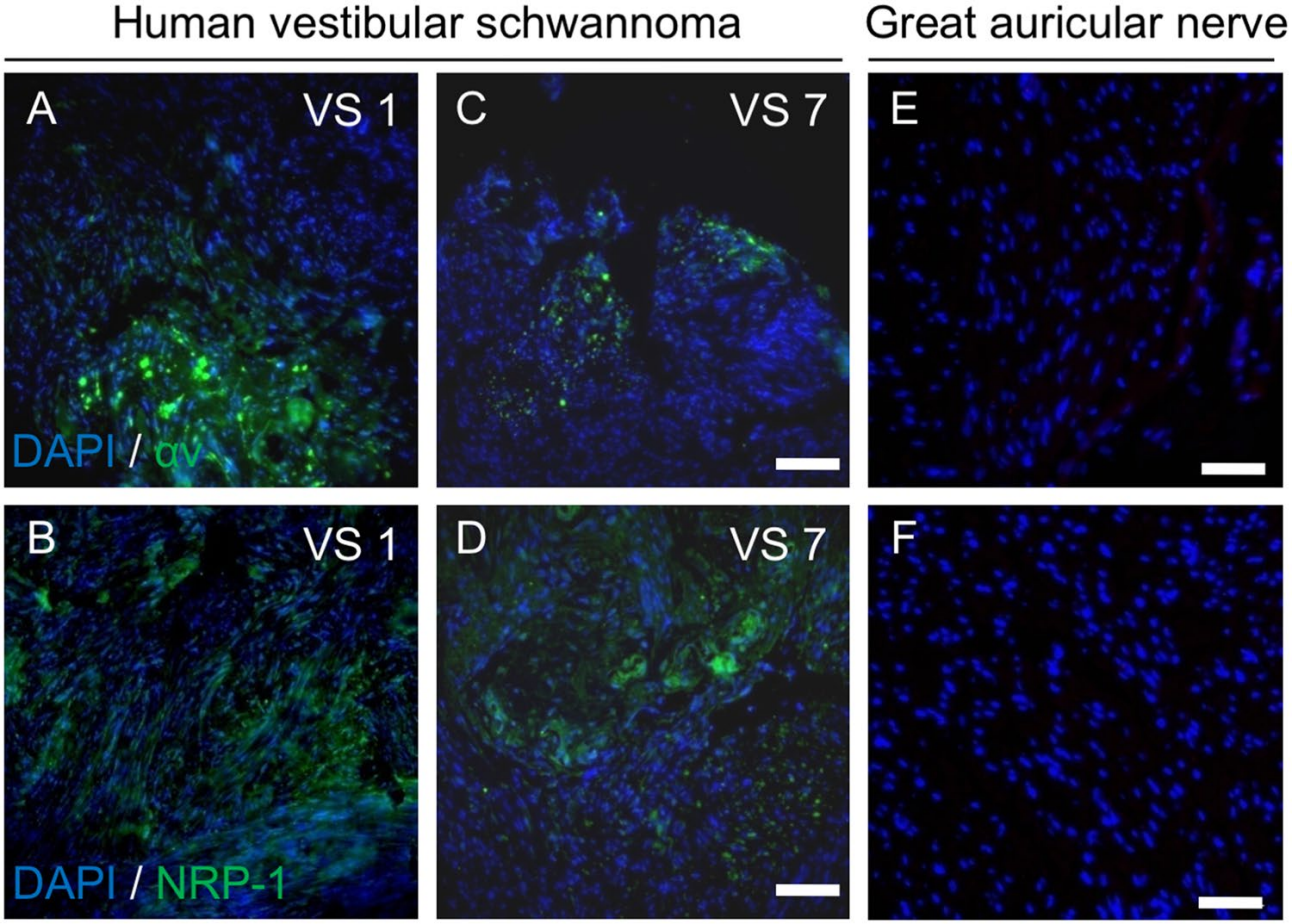
Expression of receptors for iRGD targeting in human VS specimens and healthy nerve controls. Immunofluorescence images of formalin-fixed, paraffin-embedded histological sections of human vestibular schwannomas (**A**–**D**) and great auricular nerve (GAN) controls (**E**,**F**). *Top row*, green represents αv integrin expression. Cell nuclei are counterstained with DAPI (pseudocolored﻿ blue). Scale bar, 100 μm. *Bottom row*, green represents NRP-1, blue represents DAPI nuclear stain. Scale bar, 100 μm. Representative 20x magnification images from 6–9 histological sections per specimen are shown. Specimens from 11 different VSs and 6 different GAN were analyzed.

The *in vivo* tumor targeting and tissue penetration of iRGD-directed nanoparticles is dependent on the selective presentation of receptors on the surface of tumor cells relative to surrounding neuronal tissue counterparts. To assess the potential for iRGD peptide-siRNA nanoparticles to bind to vestibular schwannoma cells *in vivo*, αv integrin and NRP-1 expression were examined in histological sections of tumors derived from patients with sporadic VSs using immunofluorescence. Control healthy nerve sections were prepared from surgically sacrificed healthy human GANs during parotidectomies and neck dissections and used as negative controls. Clusters of tumor cells that stained strongly positive for both αv integrin and NRP-1 were found throughout the tumor parenchyma (Fig. 4A–D) in 11 different tumors. Certain tumor specimens exhibited higher expression levels of both receptors, but this did not correlate with the severity of SNHL in patients (Supplementary Figure 2). By contrast, six different GAN control samples demonstrated minimal expression of either receptor (Fig. 4E,F). In sum, these results suggest that both primary human VS cultures and an NF2-derived VS cell line overexpress αv integrins and NRP-1 on the cell surface, which can be harnessed for receptor-targeted, tumor-penetrating delivery of therapeutics *via* the CendR pathway.

### Peptide-siRNA nanoparticle targeting of VS *in vitro*

**Figure 5:**
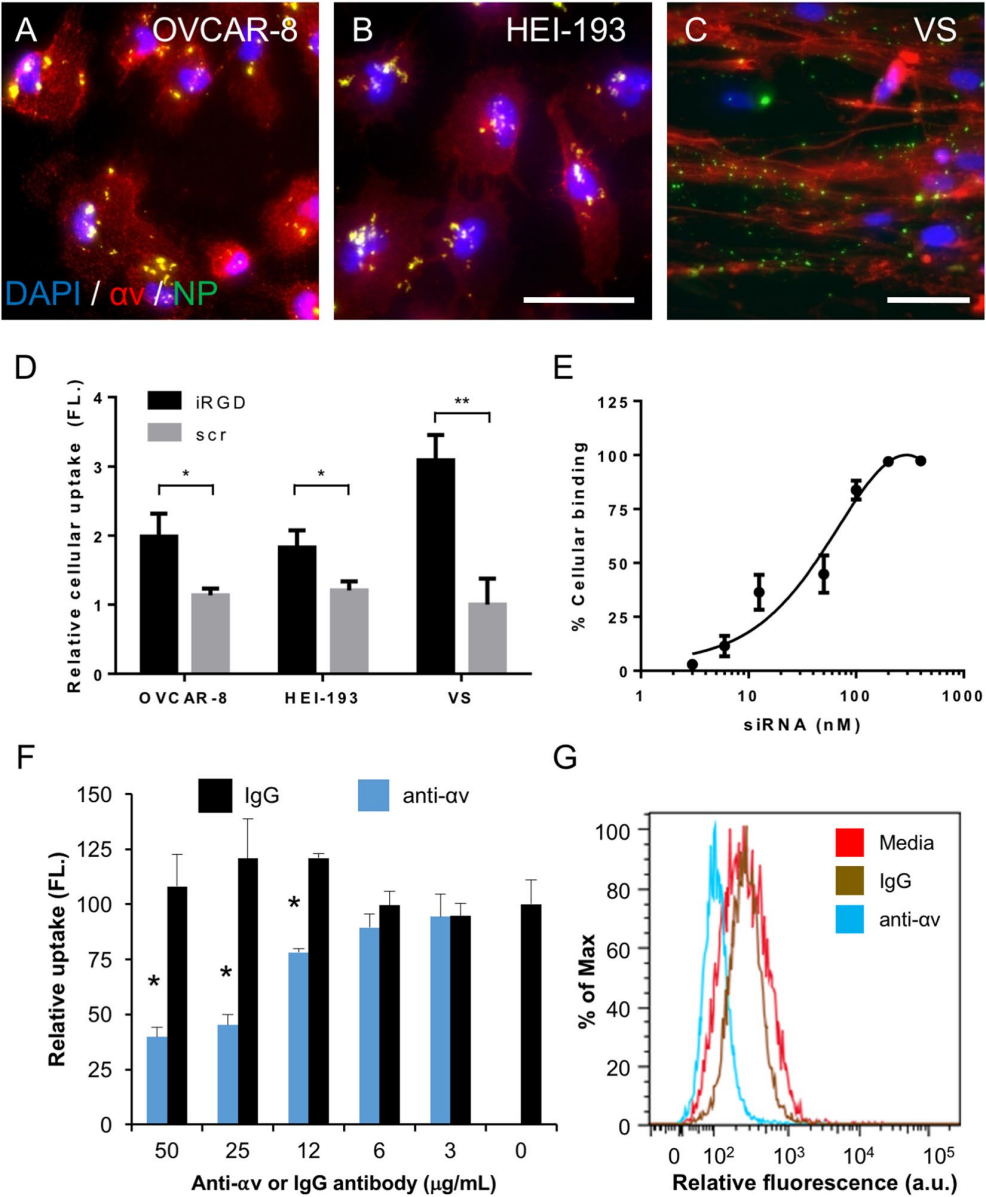
Receptor-specific targeting of iRGD nanoparticles in cancer cell lines and primary human vestibular schwannoma cells. (**A**–**C**) Representative immunocytochemistry images of nanoparticle uptake in OVCAR-8 cells (**A**, based on 6 different experiments), HEI-193 cells (**B**, based on 6 different experiments), and human vestibular schwannoma cells (**C**, based on 4 biological replicates from different patients) *in vitro*. Red, αv integrin. Green, nanoparticle (NP) with siRNA labeled with a fluorescent dye (Alexa Fluor 647). Blue, DAPI nuclear stain. Yellow pseudocolor suggests co-localization between siRNAs and αv integrin receptors. Scale bar, 100 μm. (**D**) Quantification of cellular uptake of iRGD targeted nanoparticles (iRGD, black bars) versus control nanoparticles (scr, gray bars) in OVCAR-8 cells, HEI-193 cells, and human VS cultures. Error bars represent standard deviation. N = 4–6 independent replicates. *P < 0.05, **P <0.01, *Student’s* two-tailed t-test. (**E**) Dose-dependence of nanoparticle binding in HEI-193 cells as determined by flow cytometry. Calculated K_d_ is approximately 41 nM from the binding curve. (**F**) HEI-193 cellular uptake of iRGD-targeted nanoparticles is inhibited by an antibody directed against αv integrin (blue bars) in a dose-dependent manner. A control antibody, IgG (gray bars) had no significant effect. N = 6 independent replicates. *P < 0.05, *Student’s* two-tailed t-test. (**G**) Representative flow cytometry histograms of cellular uptake of fluorescently-labeled siRNA in the presence of a blocking anti-αv integrin antibody (blue), control IgG (brown), or culture medium (red).

We next assessed the ability of iRGD-directed peptide-siRNA nanoparticles to target vestibular schwannoma cells in a receptor-specific fashion. Nanoparticles were prepared by admixing siRNA labeled with a fluorescent dye with the iRGD peptide at a molar ratio of 10:1 (peptide to siRNA). A tandem peptide bearing the non-integrin binding domain (scr) was used as a negative control to verify receptor-specificity over non-specific charge-based interactions. Independently prepared targeted iRGD and control scr nanoparticles were incubated over OVCAR-8 ovarian cancer cells, HEI-193 NF2-derived vestibular schwannoma cells, and human primary VS cells, and siRNA accumulation was assessed using both epifluorescence microscopy and flow cytometry. Compared to scr nanoparticles which displayed minimal siRNA uptake, uptake of iRGD nanoparticles by HEI-193 cells was 1.6-fold higher than scr controls at 4 °C, and 2.7-fold higher at 37 °C, likely due to contributions from the active tumor-penetration pathway (Supplementary Figure 3). Furthermore, iRGD targeted nanoparticles were strongly associated with tumor cells (Fig. 5A–C). Established cancer cell lines and primary VS cultures displayed different patterns of co-localization of fluorescent siRNAs with αv integrins. This is likely a result of differences in αv integrin surface expression, where integrin proteins localize to cell-cell contact junctions and the cellular periphery in immortalized cells but appear more diffuse in primary VS cultures. The punctate pattern of cellular accumulation at 4 °C, where receptor-mediated endocytosis pathways were likely inactive, was likely due to the clustered expression profile of αv integrins at the cellular surface. Delivered siRNA molecules tagged with a fluorophore co-localized with αv integrins but not with schwann cell marker S100, again suggesting that cellular uptake is mediated *via* the αv integrin receptor (Supplementary Figure 4). Nanoparticle targeting did not significantly affect HEI-193 cell viability in six independent experiments (Supplementary Figure 4). The amount of cellular uptake of iRGD-targeted nanoparticles was markedly higher than that of control nanoparticles, measuring approximately two-fold in established cell lines and over three-fold in human primary VS cells (Fig. 5D, Supplementary Figure 3).

If nanoparticles were indeed delivered in a receptor-dependent manner, the amount of cellular uptake of siRNA should be proportional to the dose of the ligand at low concentrations, while saturating at higher ligand concentrations as all available receptors become occupied by the ligand. To quantitatively assess the binding affinity of nanoparticles to αv integrins, HEI-193 cells were incubated with varying concentrations of iRGD-targeted nanoparticles for one hour, washed, and treated with trypsin to remove any nanoparticles loosely associated with the cell surface via non-specific interactions. Intracellular siRNA fluorescence was quantified using flow cytometry. At low nanoparticle concentrations, cellular fluorescence was linearly proportional to the amount of siRNA present, whereas at higher concentrations, cellular uptake became saturated (Fig. 5E). The apparent binding affinity as measured by the dissociation constant (K_d_) was 41 nM, consistent with values ranging from 17.8 to 61.7 nM reported in the literature^17^. To further confirm that uptake of iRGD nanoparticles was specific to the interaction between the RGD domain to αv integrins, a blocking antibody against αv was applied to cell culture prior to the addition of nanoparticles. Dose-dependent inhibition of siRNA delivery was observed with antibody concentrations ranging from 3 to 50 µg/ml, whereas the addition of a control antibody had no effect (Fig. 5F,G).

### Tumor-targeted delivery of siRNA to suppress TNFα secretion

**Figure 6:**
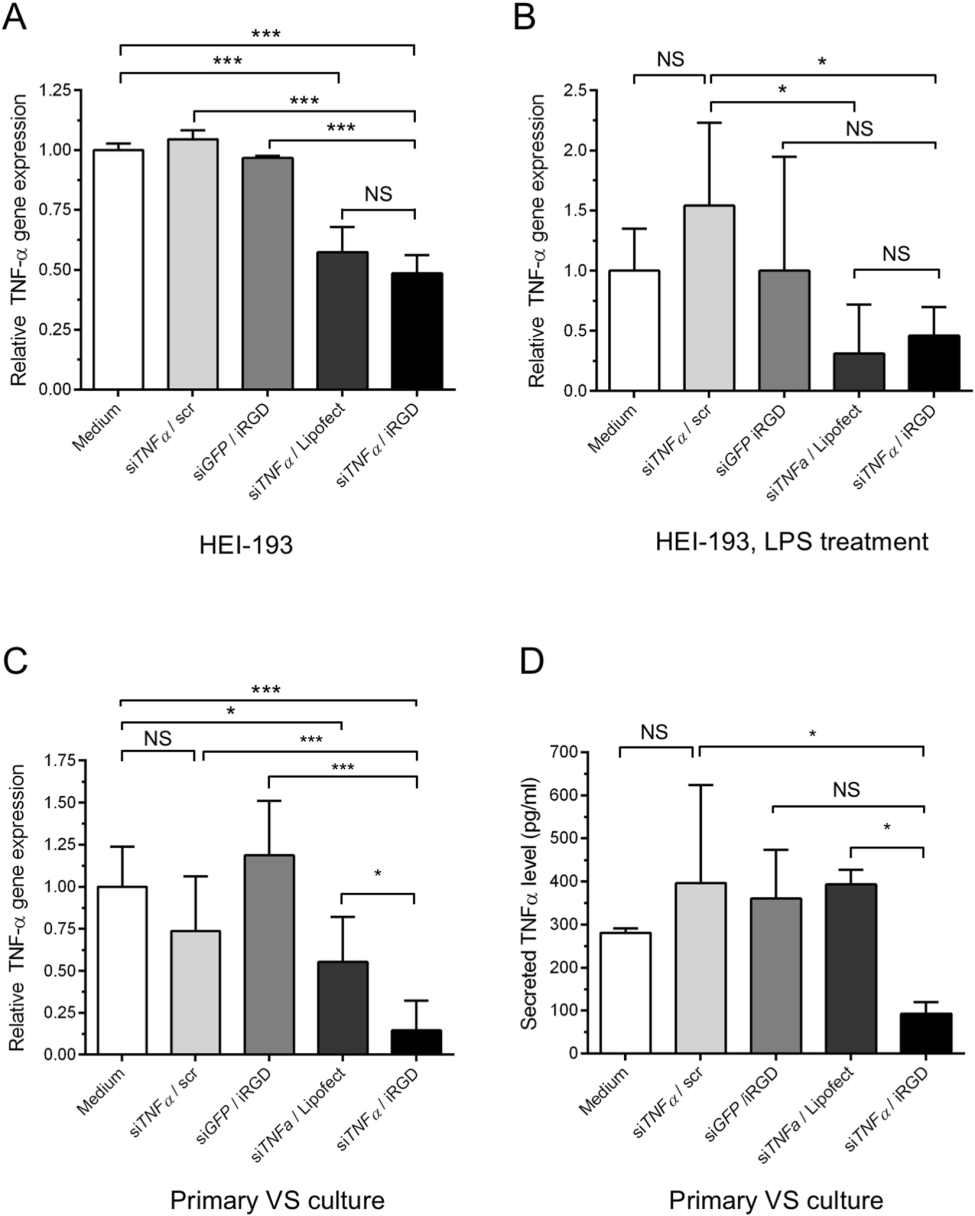
iRGD nanoparticles mediate *TNF-α* silencing by RNAi *in vitro*. (**A**) Nanoparticle delivery of *TNFα* siRNA to HEI-193 vestibular schwannoma cells. Relative *TNFα* gene expression quantified by qRT-PCR in cells treated with various formulations, in the absence of lipopolysaccharide (LPS) stimulation. Medium, untreated controls. siTNFa / scr, control nanoparticles carrying *TNFα* siRNA. si*GFP* / iRGD, iRGD nanoparticles carrying *GFP* siRNA. si*TNFα* / Lipofect, Lipofectamine RNAiMAX® complexed with *TNFα* siRNA as a positive transfection control. si*TNF*α / iRGD, iRGD nanoparticles carrying *TNFα* siRNA. Error bars represent standard deviation. N = 6 biological replicates with independently prepared nanoparticles. ***P < 0.001, one-way ANOVA. NS, not significant. (**B**) Delivery of *TNFα* siRNA to HEI-193 cells after LPS stimulation. After treatment with respective nanoparticle formulations, cells were then exposed to LPS (5 ng/mL) for 18 hours to stimulate TNFα production prior to harvesting and isolation of total RNA. Error bars represent standard deviation. N = 6 independent replicates. *P < 0.05, one-way ANOVA. NS, not significant. (**C**) Nanoparticle-mediated delivery of *TNFα* siRNA to primary human vestibular schwannomas. Relative *TNFα* mRNA levels were determined by qRT-PCR. Cells were treated with siRNA (100 nM) delivered by various formulations for 4 hours, further incubated for 48 hours, and stimulated with LPS at 5 ng/ml for 18 hours prior to harvesting. Error bars represent standard deviation. Pooled data from 4–6 different tumors are shown. NS, not significant. *P < 0.05, ***P < 0.001, one-way ANOVA. (**D**) Secreted TNFα levels from human VS after nanoparticle treatment as measured by ELISA. Error bars represent standard deviation. Pooled data from 4–6 different tumors are shown. NS, not significant. *P < 0.05, one-way ANOVA.

Having found that iRGD peptide-siRNA nanoparticles could home to VS cells *in vitro*, we next investigated the ability of nanoparticles to mediate the delivery of siRNA directed against genes of therapeutic interest. In patients with VS who also have ipsilateral SNHL, there is a positive correlation between the degree of SNHL and the amount of TNFα present in VS secretions. Application of TNFα-containing VS secretions onto murine cochlear explant cultures results in the loss and disorganization of neurites, and neutralization of TNFα partially rescued both inner and outer hair cell loss^11^. We assessed whether iRGD-targeted nanoparticles could achieve functional delivery of siRNA against TNFα to VS cells in a receptor-specific fashion. Again, nanoparticles were prepared by incubating tandem peptides bearing the pendant iRGD targeting domain with a mixture of siRNA sequences targeting non-overlapping exons of *TNFα* mRNA, whereas tandem peptides bearing the scr domain mixed with the same siRNAs were used as negative controls. Furthermore, to control for gene silencing effects that were not sequence-specific, siRNA directed against the non-human green fluorescent protein (GFP) gene was used. Various formulations of independently prepared nanoparticles were incubated over HEI-193 cells for 4 hours, and cells were harvested 48 hours later for gene expression analysis by qRT-PCR (N = 6 independent replicates per condition). Compared to cells treated with media alone, scr control nanoparticles carrying si*TNFα*, or iRGD nanoparticles carrying si*GFP*, delivery of *TNFα*-specific siRNA by iRGD-targeted nanoparticles resulted in a modest but significant suppression of *TNFα* gene expression by over 50%. By contrast, there was no statistical difference in *TNFα* gene expression between the three negative control conditions (Fig. 6A). As a positive control, transfection of si*TNFα* using a commercially available cationic lipid-based reagent (Lipofectamine RNAiMAX®) achieved approximately 40% gene silencing, which is slightly less potent than our nanoparticle formulation but does not reach statistical significance (Fig. 6A).

### Inhibition of TNFα secretion in response to pro-inflammatory stimulus

TNFα is a key cytokine produced by multiple cell types in the central nervous system in response to foreign pathogens to orchestrate an inflammatory response in many disease processes, including SNHL^33^. Therefore, we envisioned that an effective therapeutic strategy for VS should blunt the inflammatory signaling cascade by inhibiting the secretion of TNFα when cells are exposed to a pro-inflammatory stimulus. To investigate the efficacy of nanoparticle-mediated siRNA delivery, HEI-193 cells were treated with nanoparticles loaded with TNFα-specific siRNA, followed immediately by treatment with lipopolysaccharide (LPS), a known inducer of multiple cytokines as part of the inflammatory pathway^34^. All cells were treated with either medium alone or various nanoparticle formulations, followed by exposure to LPS prior to RNA collection (N = 6 independent experiments). Compared to medium-treated cells, treatment with scr control nanoparticles did not suppress *TNFα* gene expression in response to LPS. However, *TNFα* expression was significantly downregulated when siRNA against *TNFα* was introduced to the cells by either lipofectamine transfection reagent or iRGD-targeted nanoparticles. While there is a modest difference between iRGD nanoparticle treatment with si*GFP* versus si*TNFα*, this does not reach statistical significance (Fig. 6B).

Having established that iRGD-targeted delivery of siRNA can lead to receptor- and sequence-specific suppression of *TNFα* gene expression in an NF2-derived vestibular schwannoma cell line, we then evaluated the ability of iRGD nanoparticles to mediate RNAi in primary human VS cultures. Nanoparticles were again synthesized with either iRGD tandem peptides or scr tandem peptides admixed with respective siRNA cargoes. Both formulations were confirmed to exhibit similar physiochemical properties including size distributions, polydispersity, and zeta potential (Supplementary Figure 1). Nanoparticles bearing the pendant iRGD domain and si*TNFα* were applied to primary VS cultures derived from patients who also had significant ipsilateral SNHL (N = 4–6 independent tumors). After incubation with nanoparticles for 4 hours, the cells were washed and further incubated for 48 hours. Lipopolysaccharide was then added as an immunostimulatory input before tumors were harvested and tumor secretions ultimately collected for TNFα measurements 18 hours later. The relative gene expression of *TNFα* was normalized to cells that received medium alone prior to LPS exposure. VS cultures exposed to iRGD nanoparticles with *TNFα*-specific siRNA resulted in over 90% reduction in *TNFα* mRNA expression (Fig. 6C). This was statistically significant when compared to all other control conditions as well as lipofectamine treatment. Cells treated with untargeted scr nanoparticles carrying si*TNFα* demonstrated a mild decrease in *TNFα* expression when compared to medium alone, likely a reflection of siRNA uptake independent of the iRGD-αv integrin interaction. However, this difference did not reach statistical significance. VS cultures treated with iRGD nanoparticles carrying *GFP*-targeting siRNA did not show any appreciable reduction in *TNFα* expression, suggesting that gene silencing is sequence-specific. Finally, as a positive control, lipofectamine treatment led to approximately 50% reduction of *TNFα* mRNA, a less potent response than that induced by iRGD nanoparticles (Fig. 6C).

Because TNFα protein is the ultimate mediator of *ex vivo* cellular damage in murine cochlear explants^11^, we explored the ability of tandem peptide-siRNA complexes to suppress TNFα secretion after LPS challenge in VS cultures by a series of ELISAs (Fig. 6D). Primary human VSs obtained from patients with ipsilateral SNHL and high basal levels of tumor-secreted TNFα were selected for this study (N = 4–6 different tumors). After application of LPS, the mean level of TNFα in tumor secretions was between 200 to 300 pg/mL, similar to serum levels in patients with inflammatory conditions such as rheumatoid arthritis where high circulating TNFα levels were found^35^. Treatment with si*TNFα* bound to untargeted nanoparticles, *GFP*-specific siRNA in iRGD-targeted nanoparticles, or si*TNFα* mixed with a commercial transfection reagent did not lead to appreciable suppression of TNFα secretion (range, 360–396 pg/mL). By contrast, tumor-targeted delivery of si*TNFα* by iRGD tandem peptides resulted in a reduction of TNFα levels by approximately 67% to a mean level of 93 pg/mL, which was statistically significant when compared with both scr control nanoparticles carrying si*TNFα* and with lipofectamine as positive control. Collectively, these experiments show that delivery of siRNA therapeutics guided by the affinity interactions between the tumor-homing peptide iRGD and cell-surface αv integrins and neuropilin-1 resulted in potent suppression of TNFα secretion in a receptor-specific fashion.

Finally, to test the versatility and generalizability of the nanoparticle delivery system, VS cultures were treated with iRGD nanoparticles carrying siRNA directed against *RELA*, a component of the NF-κB canonical pathway implicated in VS growth and a potential therapeutic target^7^. Delivery of siRNA against *RELA* by iRGD nanoparticles resulted in significant suppression of *NF-κB1* expression, when compared to delivery of *GFP* siRNA using the same nanoparticles (Supplementary Figure 5).

## Discussion

We demonstrate for the first time that RNA interference and tumor-targeting nanotechnology can be leveraged in a synergistic fashion to deliver siRNA therapeutics to human vestibular schwannoma cells. We focus on therapeutic silencing of TNFα because of its important role as a master regulator cytokine in multiple inflammatory responses and downstream signaling pathways, including autoimmune inner ear disease^36,37^. In addition, we have previously shown that VS secretion of TNFα correlates with the severity of VS-induced hearing loss^11^. The identification of secreted factors responsible for mediating hearing loss (in addition to mechanical compression of the auditory nerve by adjacent tumor tissue and associated edema) motivates the development and validation of targeted molecular medicines, which can either be used alone or in conjunction with surgical resection and radiation therapy. Unfortunately, to date, no FDA-approved medicinal therapies exist to ameliorate or prevent VS-associated hearing loss. While monoclonal antibodies against TNFα, including Infliximab, have been used to treat autoimmune conditions such as rheumatoid arthritis, TNF-specific immunotherapies have not been deployed in VS. Moreover, the prohibitive cost of antibody development and associated autoimmunity necessitate alternative approaches.

RNAi offers an attractive approach to the perturbation and interrogation of gene targets at the mRNA level. Short interfering RNAs (siRNAs) belong to a promising class of therapeutics that could silence gene expression with high potency, specificity, and minimal side effects. In theory, RNAi can act on any gene including those not targetable by antibodies or small molecules, and is easily scalable for high-throughput screens or clinical trials. The translation of RNAi into clinical therapeutics is largely hindered by the challenge of systemic delivery of siRNA molecules^12^. When administered systemically, naked nucleic acid molecules are rapidly cleared from circulation by renal filtration. The small amount remaining in circulation is subsequently further degraded by serum nucleases. Even when siRNA is directly injected into a tumor, the negatively charged backbones prevent these molecules from crossing cellular membranes to reach the cytoplasm and engage in RISC. Delivery of siRNA against TNFα has previously been accomplished *via* direct intra-articular injection^38^, intravenous administration using liposomes or polymeric nanoparticles towards specialized cell populations in mouse models of arthritis^39,40^, and intra-oral delivery to sites of inflammation in the gastrointestinal tract for treatment of inflammatory bowel disease^41,42^. However, none of these approaches have been utilized to target tumor cell populations and cannot be generalized across disease models or tissue types.

Achieving targeted delivery of nanoparticle therapeutics to tumors has broad implications in realizing the full clinical potential of RNAi. Tumor cells and tumor-associated vasculature express unique receptors on the cell surface owing to alterations in signaling and survival pathways. Ligands such as nucleic acids^43^, aptamers^44^, and peptides^45,46^, which selectively bind to these receptors with high affinity have been harnessed to improve the cellular binding, retention, and overall efficacy of delivered compounds including siRNAs. Nevertheless, the therapeutic efficacy of macromolecular therapeutics is largely limited by their poor extravascular transport and limited tissue penetration^47^. This is likely due to a combination of abnormal blood vessels supplying the tumor, poor tissue perfusion, and high interstitial pressure due to dysfunctional tumor lymphatics^48,49^.

The targeting nanoparticle system described here offers several important advances over previously reported strategies. First, our system leverages a new class of peptides with both tumor-homing and tissue-penetrating properties. Peptides such as iRGD contain the C-terminal exposure of the C-end rule (CendR) motif (R/KXXR/K), which activates a distinct vascular transcytosis pathway to enhance the entry of macromolecular therapeutics including antibodies and nanoparticles^20,21^. The peptide selectively binds to integrins on vestibular schwannoma cells via the RGD sequence, undergoes proteolytic processing and interacts with NRP-1 via the CendR motif to trigger cellular internalization and tissue penetration. Both αv integrins and neuropilin-1 are overexpressed in numerous types of tumor tissues *in vivo*, including those of the central nervous system. Second, the tumor-targeting probe utilized here binds to its cognate receptor at high nanomolar affinity, whereas previous approaches to suppress TNF have relied on localized, lower-affinity enzymatic activity or microenvironment cues, which can be highly variable^42^. By contrast, the dissociation constant of monomeric iRGD peptide to cells bearing αv integrins is likely in the μM range, similar to that of monomeric cyclic RGD given that both peptides engage the same cell surface receptor^50^. This avidity, enhanced by approximately 20-fold, is likely due to the multivalent ligand presentation on the nanoparticle surface, thereby further reducing the off rate (K_off_)^50,51^. Third, our system is based on a “mix-and-match” paradigm, which does not require custom modification of siRNA or covalent linkage to the carrier, and is therefore readily adaptable to address other candidate genes identified from genomic screens of human schwannomas^52,53^.

The compendium of molecular markers expressed uniquely by tumor cells and tumor vasculature that can be utilized for targeting has seen an exponential expansion, owing to advances in screening technologies such as *in vivo* phage-displayed screens^54^ and gene expression profiling^55^, as well as an improved understanding of the pathophysiology of tumorigenesis and angiogenesis^56^. Correspondingly, there has been a growth in the arsenal of tumor-homing peptides to address the rising number of targets. Focusing on delivery strategies for diseases of the central nervous system, targeting of siRNA to glioma cells and neurons has been achieved using tandem peptides decorated with a transferrin-receptor targeting domain *in vitro* ^25^, and with peptides derived from rabies virus glycoprotein in a mouse model of traumatic brain injury^57^. However, neither approach enables penetration of tissue to target parenchymal tumor cells. Oncolytic recombinant herpes simplex virus (HSV) has been used to reduce tumor growth in mouse models of neurofibromatosis, but this viral vector cannot be easily modified to target specific receptors or address multiple gene targets^58^.

The nanoparticle siRNA delivery system described here has the potential to succeed in multiple areas of clinical translation. First, RNA interference is a powerful therapeutic tool to modulate gene expression that can be achieved without viral integration. Multiple clinical trials involving non-viral mediated delivery of siRNA are underway. The RNAi delivery strategy proposed here is easily scalable and readily adaptable for clinical testing. Second, unlike genes essential for tumor growth or survival for which expression is constitutively upregulated, the expression of TNFα may be more transient and associated with inflammatory stimuli. Therefore, the transient suppression of TNFα expression by RNAi may be more desirable when aiming to prevent hearing loss secondary to ototoxic secretions from vestibular schwannomas or other autoimmune etiologies. Third, our delivery system is modular and can be modified to test other genes of interest or gene combinations. As more promising targets for human vestibular schwannomas emerge, this delivery platform is poised to provide an efficient method to rapidly validate new candidate genes in primary culture systems.

The VEGF signaling axis has been implicated in orchestrating SNHL in patients with VS *via* mechanisms that are yet to be fully elucidated. Treatment with a VEGF monoclonal antibody has resulted in hearing improvement in patients with NF2-associated VS^6^, and delivery of siRNA against VEGF or HIF-1α to downregulate VEGF expression reduced glioma growth^59^. Earlier work from our laboratory has identified the transcription factor nuclear factor-kappa B (NF-κB) as a key modulator of primary VS survival, and inhibition of NF-κB by small molecules or RNAi led to reduction in cell proliferation^7^. Recently, an integrative analysis of the schwannoma genome has been completed to identify common genomic aberrations. Using a combination of whole-exome sequencing, methylation profiling and RNA sequencing, a new fusion protein that promotes tumorigenesis via the MEK-ERK signaling was found to be present in 10% of schwannomas^60^. As more promising targets begin to emerge from large-scale genomic studies, the current delivery platform can be easily adapted to perturb the expression of candidate genes in primary human VS cultures and identify those with potential to serve as therapeutic targets.

The group of subjects in this study slightly favored female patients. The role of demographic factors such as gender and age in affecting receptor expression or tumor secretions in VS has not been studied in the past. Nevertheless, some previous studies have suggested female patients may have smaller tumors and a slightly decreased incidence of hearing loss compared to males^61^. Thus, the receptor expression profile may be influenced by subjects’ gender. Further work is needed to probe demographic factors and their effects on the underlying VS tumor biology.

Looking forward, our goal is to establish a pipeline that bridges target discovery to preclinical *ex vivo* and *in vivo* validation in VS. Future studies will involve testing the efficacy and toxicity of the delivery system in xenograft and transgenic models of vestibular schwannoma. Since the delivery system is composed of modular components, simultaneous suppression of multiple gene candidates targeting distinct survival pathways can also be considered. Encouragingly, similar tandem-peptide based delivery technologies have been deployed in mouse models of brain injury with minimal toxicity^57^.

In summary, this work introduces a new approach that leverages tumor-targeting nanoparticles to improve the delivery of siRNA to vestibular schwannomas. Primary human VSs and an established schwannoma cell line both express αv integrins and neuropilin-1 on the cell surface, two receptors that serve as “zip codes” for the targeting peptide ligand iRGD. We identified a formulation of siRNA-carrying nanoparticles which home to VS in a receptor-specific manner and suppressed the pro-inflammatory ototoxic response as evidenced by reduction in TNFα secretion. As more genomic targets emerge, our delivery platform can be easily adapted to validate those genes, thereby providing a paradigm combining RNA interference and nanotechnology for preclinical validation of new therapies for vestibular schwannomas.

## Methods

### Study population and human specimen collection

Freshly harvested human VS tumor specimens from patients with sporadic VS and control great auricular nerve (GAN) were collected from indicated surgeries and transported to the laboratory in saline on ice, as is routine in our laboratory^11^. Informed consent was obtained from all patients. All study protocols were approved by the Human Studies Committee of Massachusetts General Hospital and Massachusetts Eye and Ear Infirmary, and conducted in accordance with the Helsinki Declaration. The human vestibular schwannoma cell line HEI-193, derived from a patient with neurofibromatosis type 2 (NF2), was obtained from Dr. Giovanni at the House Ear Institute.

For annotation of the patient population data, variables were defined using previously established criteria. These included: age (defined at the time of diagnosis), tumor size (largest diameter parallel to the petrous face), pure tone average (PTA; the average of the two lowest hearing thresholds in dB hearing loss measured at frequencies ranging among 0.5, 1, and 2 kHz) and word recognition (WR; the percentage of spoken words a subject can discern). Audiometric data were obtained from the latest measurements prior to tumor resection surgery. A PTA of 100 dB and WR score of 0% were assigned to a deaf ear.

### Nanoparticle synthesis

Details of peptide and siRNA synthesis and nanoparticle formulation have been described previously^22,23^. Briefly, peptides were synthesized via standard FMOC solid-phase synthesis and purified by HPLC at the MIT Biopolymers Core or CPC Scientific, Inc. The peptide sequences were: myristoyl-TP-iRGD, which targets αv integrins, CH_3_(CH)_13_GWTLNSAGYLLGKINLKALAALAKKILGGGGKC*RGDK*GPDC (Cys-Cys di-sulfide bridge, the CendR sequence is shown in italics); and myristoyl-TP-iRGE, a scrambled peptide control, CH_3_(CH)_13_GWTLNSAGYLLGKINLKALAALAKKILGGGGK C*RGEK*GPDC (Cys-Cys disulfide bridge). The purity of the peptides was confirmed by HPLC. All siRNAs were synthesized by Dharmacon (GE Healthcare) with ON-TARGETplus® siRNA design modifications to minimize off-target effects. The sequences of siRNAs were as follows (5′-3′, sense strand without overhangs): si*GFP* (GGCUACGUCCAGGAGCGCA), si*TNFα_1* (GACAACCAACUAGUGGUGC), si*TNFα_2* (GCAUGGAUCUCAAAGACAA) and si*RELA* (GGAUUGAGGAGAAACGUAA). The purity and concentration of siRNAs were confirmed with UV-spectrophotometry (NanoDrop) using absorbances at 260 nm and 280 nm. For nanoparticle synthesis, an equal volume of tandem peptide and siRNA stock aliquots prepared at 10x of the final nanoparticle concentration were used. Peptide (either iRGD or scr control) was diluted in nuclease-free ddH_2_O to a molar concentration that is 10-fold to that of siRNA, which was also diluted in nuclease-free ddH_2_O simultaneously. The two equal volumes of peptide and siRNA were admixed rapidly and allowed to form nanoparticles over 10 minutes at room temperature. The nanoparticle stock solution was then further diluted 10-fold by adding 9-part Opti-MEM (Life Technologies) to 1-part nanoparticle solution. The resulting nanoparticles at the desired concentration was then used for subsequent experiments.

### Dynamic light scattering (DLS) and zeta potential measurements

Nanoparticles were prepared by aliquoting equal volumes (10 μL) of either tandem peptide (iRGD or scr control, 50 μM) with si*GFP* (5 μM) in nuclease-free ddH_2_O at a molar ratio of 10:1 (peptide-to-siRNA) for 10 minutes at room temperature, resulting in a total volume of 20 μL. This nanoparticle stock solution was then further diluted in 80 μL of either ddH_2_O, 5% dextrose water (D5W), PBS, or Opti-MEM. For zeta potential measurements, nanoparticles were again formulated as above for a final siRNA concentration of 1 μM in 55 μL of PBS (pH 7.4) and then diluted to 800 μL with nuclease-free ddH_2_O. The hydrodynamic radii and zeta potential were determined using the ZetaSizer-Nano ZS90 instrument (Malvern). The mean particle diameter was calculated by the manufacturer’s software from the measured particle distributions.

For nanoparticle stability experiments, nanoparticles were allowed to form at room temperature as described and subsequently diluted in water, and left incubated at room temperature for 5 days prior to DLS measurement. For each zeta potential and diameter measurement, n = 5 independent preparations and characterizations were performed.

### Cell culture

Details on primary vestibular schwannoma and human Schwann cell cultures have been described previously^29,30^. Briefly, freshly harvested VS or GAN specimen was rinsed in phosphate buffered saline (PBS), dissected in culture medium consisting of Dulbecco’s Modified Eagle’s medium (DMEM) with Hams’ F12 mixture, 10% fetal bovine serum (FBS), 1% Penicillin/Streptomycin (Pen/Strep) and 1% Glutamax, all from Life Technologies. The primary VS and GAN cultures were maintained for 2 to 4 weeks as previously described^29,30^. Human ovarian cancer cell line OVCAR-8 was cultured in RPMI 1640 medium (Invitrogen) with 10% FBS, 2 mM glutamine, and 1% Pen/Strep. Human schwannoma cell line HEI-193 was cultured in DMEM with 10% FBS, 2 mM glutamine, and 1% Pen/Strep (Invitrogen).

### Antibody staining and immunofluorescence imaging

Imaging of surface expression of receptors was performed using immunocytochemistry for cell lines and primary VS cultures, and using immunohistochemistry for formalin-fixed paraffin-embedded (FFPE) tumor sections. For immunocytochemistry, live cells were rinsed in PBS, fixed in 4% paraformaldehyde (PFA) in PBS for 15 minutes, washed twice in PBS, and incubated in blocking buffer (10% goat serum, 1% BSA in PBS) for one hour. To stain for only receptors at the cell surface, the cells were not permeabilized after fixation. The cells were washed in PBS and incubated with primary antibodies against αv integrin (BioLegend, 10 μg/mL), neuropilin-1 (NRP-1, Novus Biologicals, 10 μg/mL), or S100 (Dako) diluted in 0.1% BSA in PBS overnight at 4°C. The cells were then washed in PBS three times, incubated with respective secondary antibodies (Alexa 488 goat anti-mouse or Alexa Fluor 488 goat anti-rabbit, Invitrogen, 1:500) for one hour at room temperature, washed in PBS, and counterstained with DAPI (Invitrogen) for 5 minutes, and mounted onto glass slides using Fluoromount G (Thermo Fisher). The edges of the coverslips were sealed with clear nail polish (Electron Microscopy Sciences). Images were obtained on the Nikon Ti-Eclipse TE200 inverted fluorescence microscope and NIS Elements Software (Nikon), and analyzed using ImageJ (NIH). A total of 6 biological replicates for each cell line and 3 primary VS cultures from different tumors were used for each immunostaining experiment.

For immunostaining of FFPE tumor specimens, human VS were fixed in 4% PFA and embedded in paraffin, sectioned, deparaffinized with xylene, underwent antigen-retrieval, and blocked in normal horse serum (5% NHS, 0.1% Tween-20 in PBS). The sections were incubated with primary antibodies against αv integrin or neuropilin-1, followed by corresponding fluorescent secondary antibodies. Cell nuclei were counterstained with DAPI stain (Invitrogen). The tissues were imaged using a Carl Zeiss 2000 upright microscope. Quantification of surface expression levels of αv integrin and neuropilin-1 were performed using ImageJ (NIH) on representative sections from 11 different tumors. For each tumor specimen, at least six to nine randomly selected histological sections were chosen for quantification. For great auricular nerve staining, 6 distinct patient-derived specimens were used.

### Flow Cytometry

Fluorescence-activated cell sorting (FACS) analyses of αv integrin and neuropilin-1 expression were performed on OVCAR-8, HEI-193, and primary VS cells. Briefly, OVCAR-8 and HEI-193 cells were passaged in enzyme-free cell dissociation buffer and allowed to grow to 70% confluence prior to harvesting. The cells were trypsinized using enzyme-free cell dissociation solution and brought to single-cell suspension in 1% BSA in 1 × PBS on ice. Approximately 0.5 x 10^6^ cells were used for each condition. Primary antibody was added at 1 μg per 1 × 10^6^ cells and incubated for one hour on ice. For integrin staining, mouse anti-human αvβ3 antibody (BioLegend) was used. For neuropilin-1 staining, rabbit anti-NRP-1 (Novus Biologicals) was used. Cells were washed in PBS and incubated with fluorescently-tagged secondary antibodies (Alexa 488 goat anti-mouse and Alexa Fluor 647 goat anti-rabbit, Invitrogen, 1:1000) for one hour on ice. Cells were then washed in PBS twice, re-suspended in 1% BSA in PBS containing 1:2000 PI and analyzed on the BD LSRII flow cytometer. Six (6) independent replicates for each cell type were performed. For primary VS samples, cells from 4 different tumors were pooled to ensure sufficient number of cells for flow cytometry. Data was analyzed in FlowJo (TreeStar Software).

### Cellular uptake of nanoparticles

For cellular uptake experiments, both established cell lines (OVCAR-8 human ovarian cancer cells or HEI-193 human vestibular schwannoma cells) were passaged in 1x enzyme-free cell-dissociation solution (EMD Millipore) 48 hours prior to allow regeneration of cell surface receptors, and grown in 24-well tissue culture plates on glass coverslips to 50–70% confluence. Primary VS cultures were grown as described previously. On the day of the experiment, nanoparticle formulations were prepared at indicated concentrations diluted in Opti-MEM. For visualization by immunofluorescence, siRNA molecules pre-labeled with Alexa Fluor 647 (Dharmacon) were used with either iRGD or scr control peptides. The cells were washed with PBS prior to incubation with nanoparticles. Freshly prepared nanoparticles were added to cells at 4 °C for 45 minutes for assessment of cellular binding, or at 37 °C for 45 minutes if assessing internalization. The nanoparticle solutions were then gently aspirated and cells were rinsed in PBS twice, and processed for immunocytochemistry as described below. For flow cytometry, cells were washed with PBS, trypsinized using 0.025% trypsin to remove non-specifically bound nanoparticles, centrifuged to cell pellet at 300g for 5 minutes, and re-washed with 1% bovine serum albumin (BSA) in PBS at 4 °C two more times. The cells were finally re-suspended in 1% BSA in PBS with 1:2000 Propidium Iodide (PI; Invitrogen). The cells were analyzed for siRNA fluorescence gating PI-negative cells (BD LSR II instrument). For each experiment involving established cell lines, six (6) independent replicates with separately prepared nanoparticles and cell cultures were used and results were averaged. For each experiment involving primary VS cultures, four (4) distinct VS cultures from different patients were used and data was pooled.

To examine the specificity of receptor targeting, a competition assay with anti-αv integrin antibody was used to block the RGD-integrin interaction. HEI-193 cells were passaged in enzyme-free dissociation buffer and grown in 96-well plates to approximately 50–70% confluence for 48 hours, as described previously. On the day of the experiment, the cells were washed with PBS and pre-incubated with either αv-integrin antibody or isotype-control IgG antibody in Opti-MEM at indicated concentrations from 3 μg/mL to 50 μg/mL for 1 hr. Nanoparticles were then added to a final concentration of 100 nM siRNA and uptake was assessed as described. Results were reported from 6 independent replicates.

### Assessment of cell viability

HEI-193 cells were plated in 96-well plates at 10,000 cells per well and allowed to grow to 50–70% confluence over 48 hours (N = 6 independent replicates). The cell culture medium was removed and si*GFP* complexed to either iRGD tandem peptides or scr control peptides were added to cells in Opti-MEM for 4 hours. After incubation with nanoparticles, 50 μl of the medium was removed and 50 μl of CellTiter-Glo reagent was added to each well. The plate was placed on an orbital plate shaker for 2 minutes at room temperature for cell lysis, and 80 μl of the cell lysate was transferred to a white-bottomed 96-well plate and luminescence was measured on the Tecan SpectroMax Gemini microplate reader (Molecular Devices).

### Delivery of siRNA in cell culture

For TNFα silencing, iRGD or scr control nanoparticles loaded with si*TNFα* or si*GFP* were prepared independently, applied to HEI-193 cells or primary VS cultures in antibiotic-free Opti-MEM for 4 hours, followed by wash in PBS and exchange to serum-containing DMEM for an additional 2 days prior to harvesting of either total RNA or cell-conditioned media. A commercial lipid transfection reagent, Lipofectamine RNAiMax (Invitrogen), was used as a positive control according to manufacturer’s instructions. For experiments involving Lipopolysaccharide (LPS) treatment, LPS stock solutions (Sigma-Aldrich) were prepared in DMSO and frozen at −80 °C until further use. After treatment with various nanoparticle formulations and controls, LPS was added to cells at a final concentration 5 ng/ml for 18 hours at 37 °C, washed twice with PBS, and RNA or cell-conditioned media were collected as published previously by our laboratory^11^. For knockdown experiments in HEI-193 cells, six independent biological replicates were used. For experiments in primary VS cultures, a total of 4–6 independently prepared nanoparticle formulations on cultures derived from different patients were used.

For RNAi inhibition of the NF-κB signaling pathway, nanoparticles were formulated with siRNAs against *RELA* or a control gene at a molar ratio of 10:1 (peptide to siRNA), per the protocol described above. Lipofectamine was used as a positive control. Primary VS cultures (N = 3 different tumors) were washed in PBS and incubated with various nanoparticle formulations in serum-free Opti-MEM for 4 hours at 37 °C, followed by exchange to serum-containing DMEM for an additional 2 days prior to harvesting of total RNA. The efficiency of gene knockdown was quantified using real-time quantitative PCR.

### Quantitative RT-PCR

Quantitative real-time PCR (qRT-PCR) was performed with an Applied Biosystems Step One Plus Real-time PCR instrument using SYBR green PCR Master Mix (Qiagen). Total RNA was extracted with Trizol (Invitrogen) and used to synthesize the first-strand cDNA by using Oligo (dT)_20_ /random hexamer primer cocktails and SuperScript III reverse transcriptase (Invitrogen). For the PCR reaction, per each well of 25 μl final volume, there was 12.5 μl 2X SYBR Green qPCR Master Mix (Applied Biosystems), 10.5 μl of ddH2O, 1.0 μl of cDNA template (up to 100 ng of cDNA), and 1.0 μl (10 μM) of paired PCR primers. The thermal cycler profile was: Cycle 1: 95 °C for 10 min; Cycle 2: 40 cycles of 95 °C for 10 sec and 60 °C for 60 sec. The primer sequences were as follows: TaqMan Primers (Applied Biosystems) for *NFKB1* (encoding p50 subunit of the NF-kB heterodimer, Hs01042010_m1), *RELA* (encoding p65 subunit of the NF-kB heterodimer, Hs01042010_m1), *TNF* (Hs01042010_m1), and GAPDH. Triplicate reactions for the gene of interest and the endogenous control were performed separately on the same cDNA samples, and each condition represents data from 6 independent replicates for HEI-193 cells, and 4 to 6 distinct tumors. For data analysis, the melt curves were verified and only curves with one melting peak were used. GAPDH housekeeping gene was used as the endogenous control. C_T_ values for genes were geometrically averaged and used for ΔΔC_T_ calculations. Specifically, the relative fold change in gene expression was calculated as 2^−Δ(ΔCT)^, where ΔC_T_ = C_T, target_ − C_T, GAPDH_, and ΔΔC_T_ = ΔC_T, treatment_ − ΔC_T, control_.

### Measurement of TNFα secretions

Following nanoparticle treatment and LPS stimulation, the tumor cell secretions were collected in DMEM in triplicate by removing 100 μl of the conditioned media. All secretions were then snap frozen and stored at −80 °C until further use. The concentrations of TNFα protein from untreated tumor cells, cells treated with nanoparticles carrying *TNFα* siRNA or control siRNA, scr control nanoparticles carrying *TNFα* siRNA, or lipofectamine positive controls were quantified by using human TNFα ELISA kit (R&D) following an established protocol^11^. A total of 4–6 independently prepared nanoparticles on unique primary VS cultures from different patients were used for each condition.

### Statistical analyses

Unless otherwise indicated, one-way ANOVA was used (GraphPad) and *P* < 0.05 was considered statistically significant. Tukey’s multiple comparison test was performed in ANOVA analysis. Two-tailed Student’s *t* test was used for pairwise comparisons.

## Electronic supplementary material

Supplementary Materials
