# Supplementary material for: Tumor-Penetrating Delivery of siRNA against TNFα to Human Vestibular Schwannomas
Source: Sci Rep. 2017 Oct 10;7:12922. doi: 10.1038/s41598-017-13032-9 (PMC5635039; doi:10.1038/s41598-017-13032-9)
Supplement: Supplementary file 1 — Supplementary Materials [file 41598_2017_13032_MOESM1_ESM.pdf]

TITLE: Tumor-Penetrating Delivery of siRNA against TNFα to Human Vestibular Schwannomas

AUTHORS: Yin Ren, Jessica E. Sagers, Lukas D. Landegger, Sangeeta N. Bhatia, and Konstantina M. Stankovic

SUPPLEMENTARY MATERIALS

Includes Supplementary Figures 1-5

Supplementary Figure 1

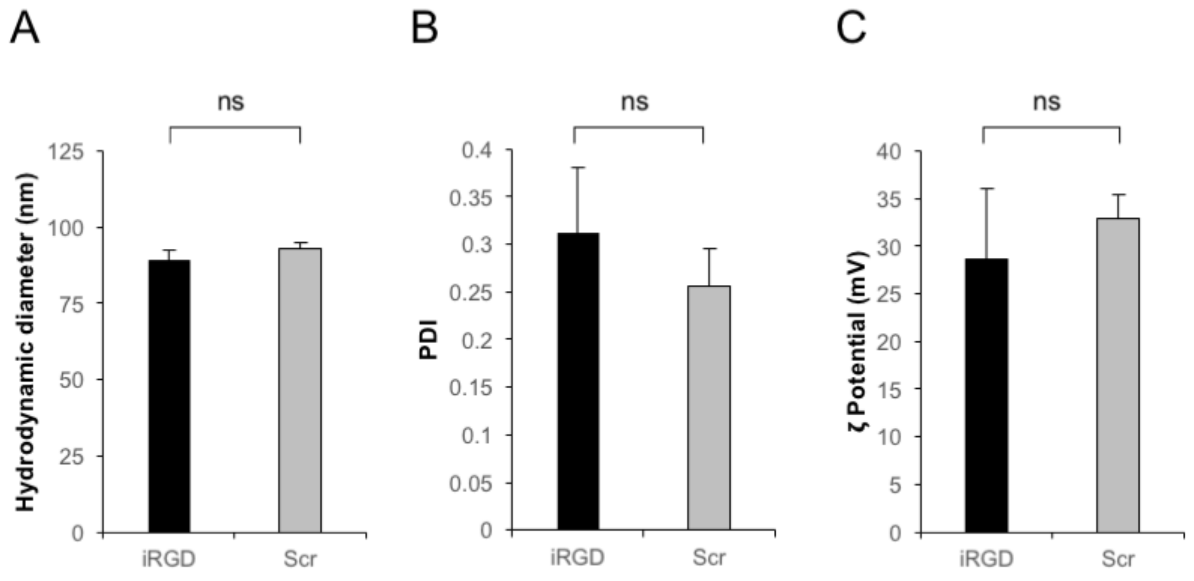

Supplementary Figure 1. Physiochemical characterization of targeted (iRGD) and untargeted control (scr) nanoparticles. **A.** Hydrodynamic diameter of iRGD (black bars) versus scr (gray bars) nanoparticles. **B.** Polydispersity index (PDI) of iRGD versus scr nanoparticles. **C.** Zeta potential (mV) of iRGD versus scr nanoparticles. N = 5 independent nanoparticle preparations for each measurement. All error bars represent standard deviation. Ns, not significant.

Supplementary Figure 2

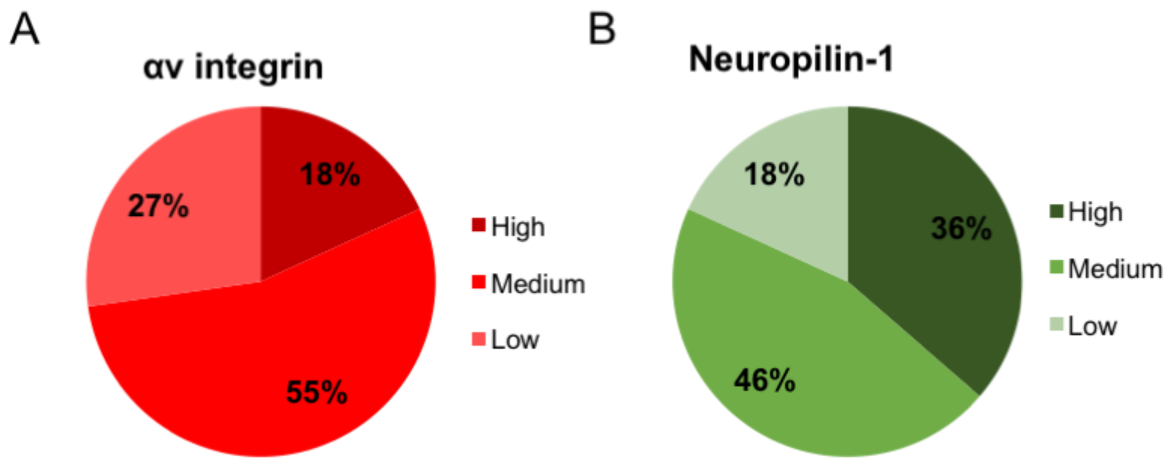

Supplementary Figure 2. Quantification of αv integrin and neuropilin-1 (NRP-1) surface expression in representative histological sections of human VS. N = 11 different tumors were analyzed.

Supplementary Figure 3

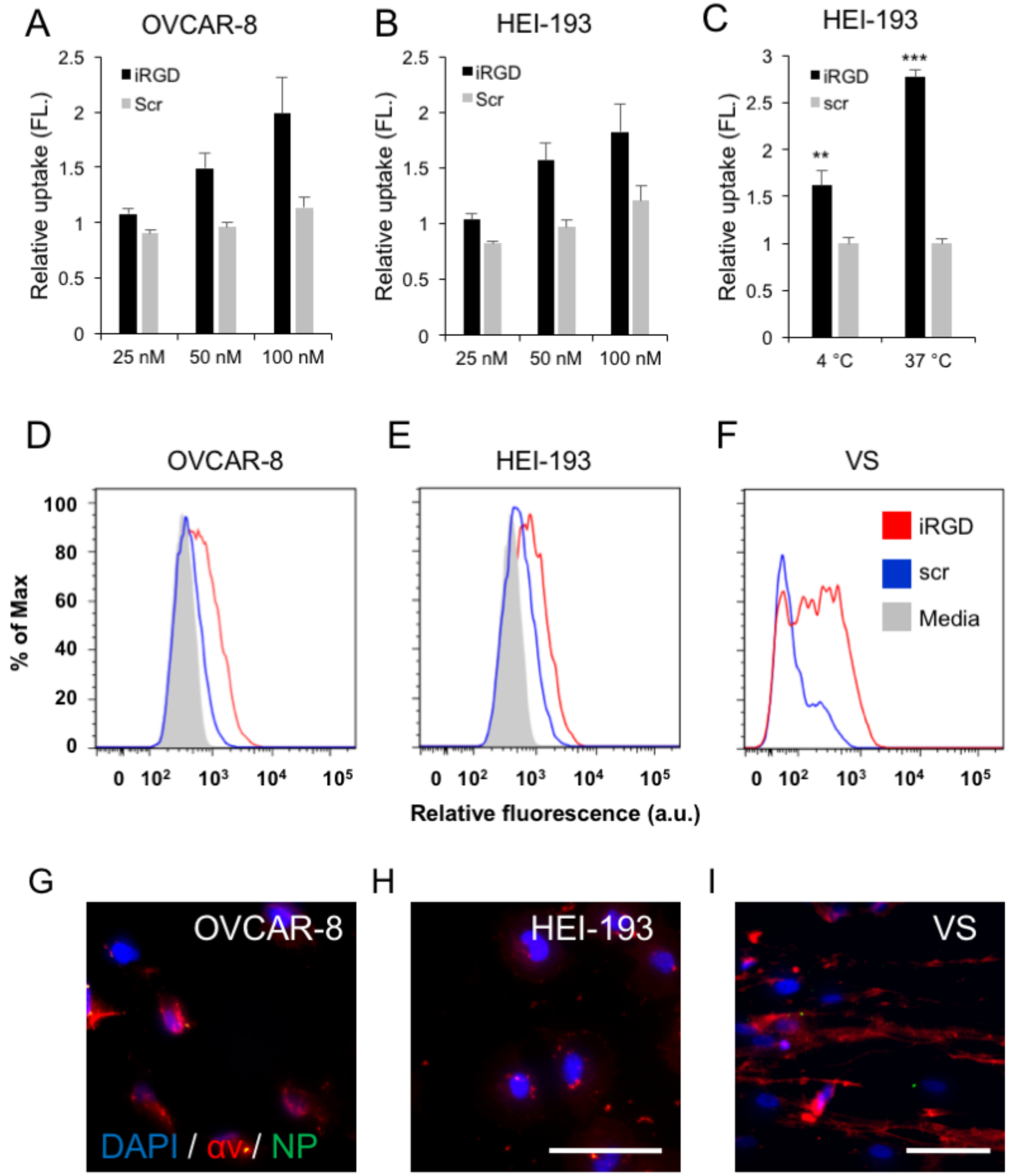

Supplementary Figure 3. Cellular uptake of nanoparticles is both dose-dependent and receptor-specific. **A-B.** Uptake of iRGD (black bars) vs. scr nanoparticles (gray bars) in OVCAR-8 (*A,*

based on 6 different experiments) and HEI-193 (*B,* based on 6 different experiments) cells at varying concentrations as measured by flow cytometry. **C.** Cellular uptake of iRGD versus scr nanoparticles at 4 °C and at 37 °C. N = 6 independent replicates. Error bars represent standard deviation. $^{**}P < 0.01$, $^{***}P < 0.001$, *Student's* two-tailed t-test. **D-F.** Representative histograms of nanoparticle uptake. Red, iRGD nanoparticles; Blue, scr control nanoparticles; Gray, cells treated with media alone. **G-I.** Representative immunocytochemistry of scr nanoparticle uptake in OVCAR-8 (*G,* based on 6 different experiments), HEI-193 (*H,* based on 6 different experiments), and human vestibular schwannomas (*I,* based on 4 different tumors) *in vitro*. Red, αv integrin. Green, Nanoparticle (NP) with siRNA labeled with a fluorescent dye (Alexa Fluor 647). Blue, DAPI nuclear stain. Scale bar, 100 μm.

Supplementary Figure 4

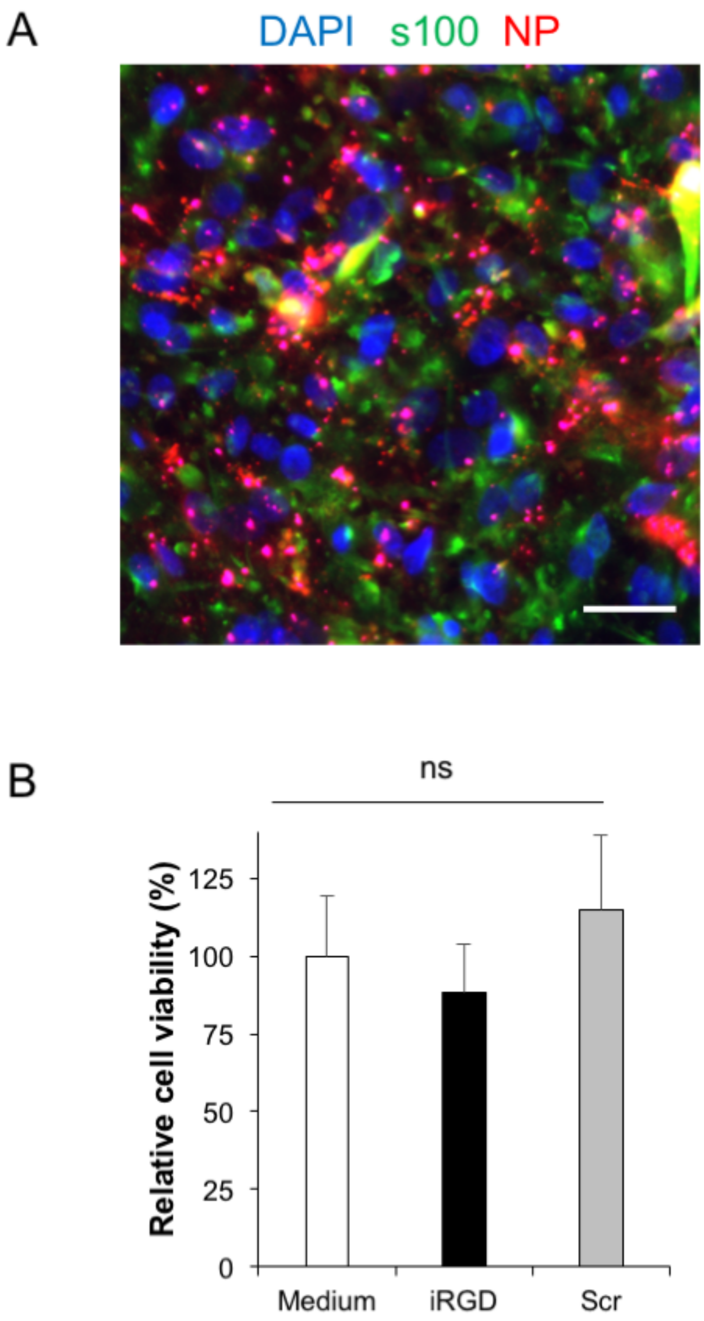

Supplementary Figure 4. Nanoparticle uptake and cell viability. **A.** Representative immunofluorescence image of nanoparticle uptake in human vestibular schwannoma cultures. The schwann cell marker s100 is pseudocolored green and nanoparticle is colored red. Nuclei are

counterstained in DAPI (blue). Scale bar, 100 μm. **B.** Relative cell viability of HEI-193 cells treated with iRGD or scr nanoparticles (100 nM), normalized to media-treated cells. N = 6 independent experiments. Error bars represent standard deviation. Ns, not significant by one-way ANOVA.

Supplementary Figure 5

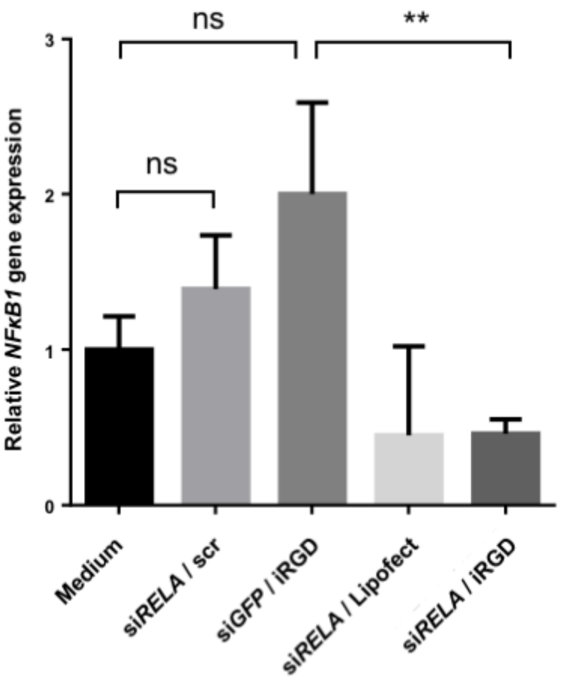

Supplemental Figure 5. iRGD nanoparticles delivers *NF-κB* siRNA to human vestibular schwannomas *in vitro*. Nanoparticle mediated delivery of siRNA against *RELA (p65)* in primary human VS cultures. *NF-κB1* gene expression levels are quantified by qRT-PCR. Error bars represent standard deviation (N = 3 biological replicates with independently prepared nanoparticles). NS, not significant. $^{**}P < 0.01$, one-way ANOVA.
